# Exosomal microRNAs in Bronchial Aspirate and Other Liquid Biopsy Specimens for Lung Cancer: Current Evidence and Future Perspectives—A Narrative Review

**DOI:** 10.3390/cells15080731

**Published:** 2026-04-20

**Authors:** Dragoș Huțanu, Mara Andreea Vultur, Corina Eugenia Budin, Dumitru Cătălin Sârbu, Maria Beatrice Ianoși, Edith Simona Ianoși, Hédi Katalin Sárközi, Gabriela Jimborean

**Affiliations:** 1 Pulmonology Department, George Emil Palade University of Medicine, Pharmacy, Science, and Technology of Târgu Mureș, 540139 Târgu Mures, Romania; dragos.hutanu@umfst.ro (D.H.); mara.vultur@umfst.ro (M.A.V.); edith.ianosi@umfst.ro (E.S.I.); hedi.balog@umfst.ro (H.K.S.); gabriela.jimborean@umfst.ro (G.J.); 2 Doctoral School of Medicine and Pharmacy, George Emil Palade University of Medicine, Pharmacy, Science, and Technology of Târgu Mureș, 540139 Târgu Mureș, Romania; 3 Pulmonology Clinic, Mureș County Clinical Hospital, 540011 Târgu Mureș, Romania; ianosi.maria-beatrice@stud18.umfst.ro; 4 Pathophysiology Department, “George Emil Palade” University of Medicine, Pharmacy, Science and Technology of Târgu Mureș, 540139 Târgu Mureș, Romania; corina.budin@umfst.ro

**Keywords:** lung cancer, micro-ARN, exosomes, cancer

## Abstract

Lung cancer remains the leading cause of cancer mortality worldwide, with most cases diagnosed at advanced stages. Conventional tissue biopsy is invasive, and low-dose CT (LDCT) screening—although effective—faces practical and logistical limitations. Liquid biopsy has emerged as a minimally invasive approach to capture tumor-derived material, including circulating tumor DNA (ctDNA), cells, and extracellular vesicles (EVs). Among EVs, exosomes and their microRNA (miRNA) cargo offer a stable, disease-specific signal. Airway-proximal fluids such as bronchial aspirate and bronchoalveolar lavage fluid (BALF) are in direct contact with the tumor microenvironment and may contain higher concentrations of tumor-derived exosomal miRNAs compared with blood. This review synthesizes the limited but promising evidence for exosomal miRNAs in bronchial aspirate and BALF as diagnostic and prognostic biomarkers in lung cancer, examines methodological and standardization challenges, and discusses potential integration into clinical workflows, with particular emphasis on Romania’s lung cancer epidemiology and healthcare context. While only two primary studies in the last five years have explored BALF exosomal miRNAs, these data justify further multicenter investigations aligned with MISEV2023 guidelines. Integrating airway-proximal exosomal miRNA analysis into bronchoscopy procedures could enhance diagnostic precision in resource-limited health systems and support the transition towards personalized thoracic oncology.

## 1. Introduction

Lung cancer remains the leading cause of cancer mortality globally, despite major advances in imaging and therapy. Most cases are still diagnosed at advanced stages, limiting curative potential. The GLOBOCAN 2022 report estimates over 2.2 million new cases and 1.8 million deaths annually, corresponding to nearly one in five cancer deaths worldwide [1].

In Romania, lung cancer continues to represent the most lethal malignancy, with over 10,000 deaths in 2022 and a lifetime mortality risk of approximately 3.3%, among the highest in the European Union [2]. National and European reports (OECD/EC, EU Country Cancer Profile: Romania 2025) show that despite lower overall cancer incidence, mortality rates in Romania remain above EU averages, particularly among men, due to late-stage diagnosis, limited screening coverage, and uneven access to advanced diagnostics [3]. Moreover, analyses of lung-cancer epidemiology in Romania point to an increasing incidence in both sexes, driven by tobacco use and environmental exposures [3,4].

Romania’s National Plan for Beating Cancer 2023–2030, aligned with the National Health Strategy 2023–2030, aims to strengthen prevention, screening, and oncology infrastructure [4]. However, population-level LDCT screening remains in pilot phases, and diagnostic delays persist, highlighting the need for complementary biomarkers to enable earlier detection and stratification.

### 1.1. Low-Dose CT Screening and Diagnostic Limitations

Two landmark trials—the National Lung Screening Trial (NLST) and NELSON—demonstrated that annual LDCT reduces lung cancer mortality by approximately 20–26% in high-risk populations [5,6]. Despite this, widespread implementation faces challenges: high false-positive rates, overdiagnosis, cost, and limited capacity for imaging follow-up. Therefore, adjunct biomarkers capable of refining nodule characterization and triaging patients are urgently needed [7].

### 1.2. The Liquid Biopsy Paradigm

Liquid biopsy—the analysis of tumor-derived material in biofluids—includes circulating tumor DNA (ctDNA), circulating tumor cells (CTCs), and extracellular vesicles (EVs). EVs, particularly exosomes, encapsulate nucleic acids and proteins reflective of the tumor phenotype and are detectable in plasma, serum, sputum, pleural fluid, and airway-proximal fluids such as bronchial aspirate and bronchoalveolar lavage fluid (BALF) [8].

**Figure 1:**
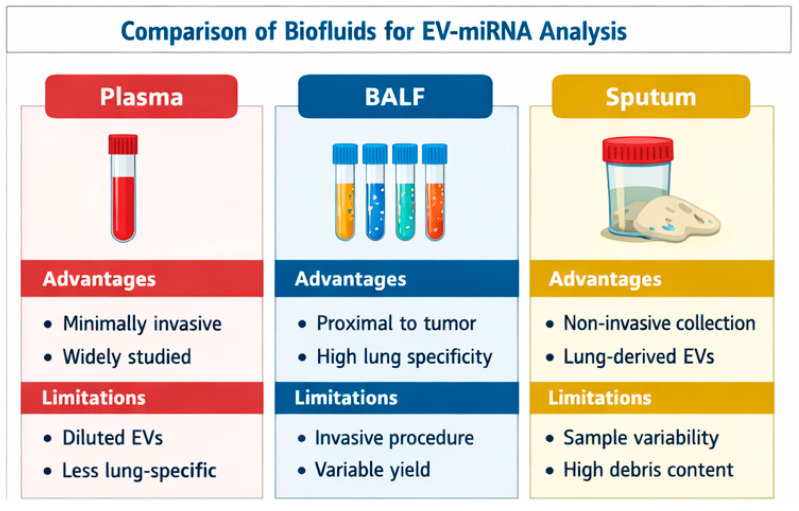
Comparison of biofluids for EV-miRNA analysis.EV–extracellular vesicles; BALF–bronchoalveolar lavage fluid;.

Compared with plasma, BALF and bronchial aspirate may capture more localized tumor signals, offering diagnostic advantages in early or localized disease. This proximity underlies growing interest in exosome-based biomarkers from airway samples [9] (Figure 1). 

Importantly, this review focuses on a highly specific and underexplored domain of liquid biopsy—airway-derived extracellular vesicle-associated microRNAs—where current evidence remains limited but biologically compelling. By synthesizing available data and emphasizing translational perspectives, this work aims to provide a focused framework for future clinical validation.

## 2. Biological Background: Extracellular Vesicles, Exosomes, and miRNAs

**Figure 2:**
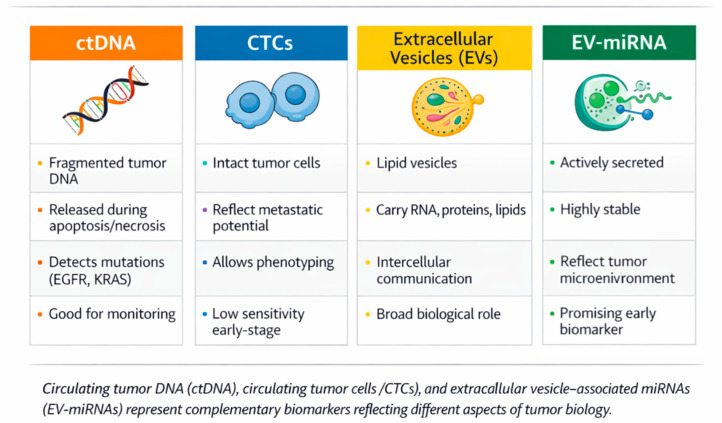
Comparative overview of major liquid biopsy components in lung cancer.

Liquid biopsy encompasses multiple classes of tumor-derived biomarkers, each reflecting distinct biological aspects of cancer (Figure 2). Circulating tumor DNA (ctDNA) provides insights into tumor genomics and mutational profiles, enabling detection of actionable mutations and monitoring of minimal residual disease. Circulating tumor cells (CTCs) offer a cellular perspective, allowing phenotypic and functional characterization of tumor dissemination, although their detection remains technically challenging in early-stage disease [9].

In contrast, extracellular vesicles (EVs), which are nano-sized, membrane-bound particles released by most cells, including exosomes, are actively secreted by tumor and stromal cells and carry a diverse cargo of nucleic acids, proteins, and lipids. Among these, EV-associated microRNAs (miRNAs) have emerged as particularly promising biomarkers due to their stability, abundance, and ability to reflect dynamic tumor-host interactions. Unlike ctDNA, which is primarily released during cell death, EV-miRNAs may capture ongoing biological processes such as immune modulation, angiogenesis, and metastatic signaling, providing complementary information for diagnosis and disease monitoring. [10,11]

In lung cancer, exosomal microRNAs (miRNAs)—small, non-coding RNAs that regulate gene expression—act as oncogenic or tumor-suppressive molecules. Their encapsulation within lipid bilayers confers extraordinary stability in biological fluids, making them suitable for liquid biopsy [12].

### Standardization and Reporting

**Figure 3:**
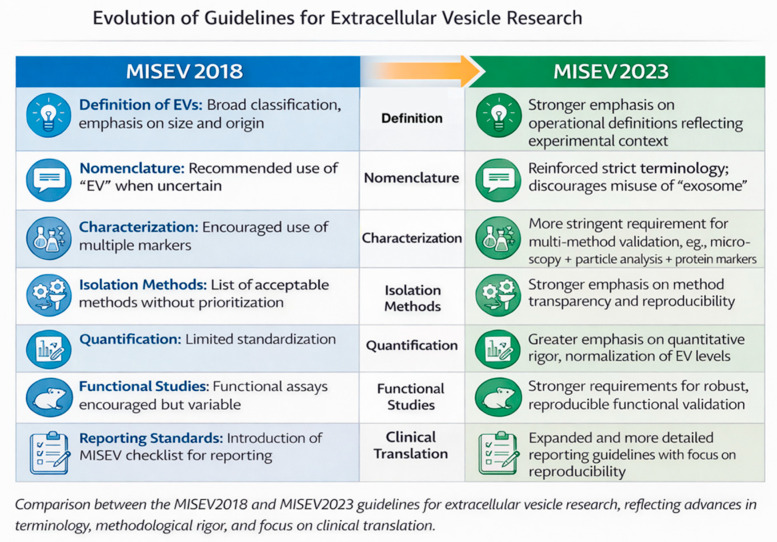
Evolution of Guidelines for Extracellular Vesicle Research.

To ensure reproducibility, the International Society for Extracellular Vesicles (ISEV) developed the MISEV2018 and updated MISEV2023 guidelines, which define minimal standards for EV isolation, quantification, and reporting [13,14]. These guidelines stress the importance of using multiple complementary isolation and characterization methods (e.g., nanoparticle tracking, immunoblotting, electron microscopy) to confirm exosome identity (Figure 3). Adherence to these standards is especially crucial for airway-derived samples, which vary in viscosity, mucin content, and cellular debris that can compromise EV purity [15].

## 3. Liquid Biopsy in Lung Cancer: Positioning EV-miRNAs

Liquid biopsy is now recognized as a cornerstone of precision oncology. While circulating tumor DNA (ctDNA) and circulating tumor cells (CTCs) are well studied, their sensitivity in early-stage lung cancer remains suboptimal [16]. By contrast, exosomal miRNAs are actively secreted and may capture dynamic tumor processes independent of cell turnover [17,18].

Comprehensive reviews have documented that exosomal miRNAs such as miR-21, miR-126, and let-7a show high diagnostic potential in plasma and BALF samples [19,20]. Among all matrices, BALF and bronchial aspirate stand out for their proximity to tumor tissue, conferring higher specificity but lower scalability due to procedural requirements [21].

## 4. Methods Landscape for Airway-Proximal Sampling

BALF collection during bronchoscopy involves instilling sterile saline (20–100 mL) into a targeted bronchopulmonary segment and aspirating the recovered fluid [22]. Bronchial aspirate or washing samples, obtained without instillation, reflect proximal airway secretions and are simpler to collect [23]. These specimens must be processed rapidly, ideally within two hours, to preserve vesicle integrity [24].

Isolation techniques include ultracentrifugation, polymer precipitation, and size-exclusion chromatography (SEC), each with trade-offs between yield and purity [25]. Immuno-capture and microfluidic EV isolation platforms are emerging as clinical-grade alternatives due to scalability and reproducibility [26,27]. RNA extraction and quantification rely on qPCR, digital PCR, or small-RNA sequencing, but normalization across airway matrices remains a key challenge [28].

## 5. Evidence Summary: Exosomal miRNAs in BALF and Bronchial Aspirate

### 5.1. Evidence Base and Literature Synthesis

This manuscript is conceived as a narrative review focusing primarily on original studies that investigate exosomal or extracellular vesicle-associated microRNAs (miRNAs) in airway-proximal fluids—specifically bronchoalveolar lavage fluid (BALF) and bronchial aspirate—in the context of lung cancer. Given the emerging nature of this field, no formal meta-analyses dedicated exclusively to airway-derived exosomal miRNAs in lung cancer are currently available. Therefore, evidence synthesis relies on a small number of exploratory and translational original studies, complemented by high-quality narrative reviews and methodological position papers to provide biological and technical context.

The literature discussed in this review was identified through targeted searches of PubMed/Medline and Scopus, focusing on publications from approximately 2015 to 2024, a period that corresponds to the maturation of extracellular vesicle research and the introduction of standardized reporting frameworks (MISEV2018 and MISEV2023) [13,14]. Earlier studies were included selectively when they represented proof-of-concept work or foundational observations. The cited evidence originates from international cohorts, including studies conducted in Europe and East Asia, reflecting the global but still limited research activity in this niche area. No region-specific restriction was applied, as the total number of eligible studies remains small.

Most liquid biopsy research in lung cancer has focused on blood-based biomarkers, particularly circulating tumor DNA and plasma exosomal miRNAs. In contrast, airway-proximal fluids have been comparatively underexplored due to procedural requirements and lack of standardization. By explicitly concentrating on BALF and bronchial aspirate, this review addresses a distinct biological compartment that is in direct contact with the tumor microenvironment and may offer higher tumor specificity, particularly in early or localized disease.

The limited volume of available literature underscores that airway-derived exosomal miRNA analysis remains an innovative and investigational approach. Only a few original studies over the past decade—and only two within the last few years—have directly examined exosomal miRNAs in BALF in lung cancer. This scarcity justifies the emphasis on future directions, methodological harmonization, and translational potential rather than definitive clinical conclusions. Highlighting innovation and unmet needs is therefore central to the structure of this review.

### 5.2. Key Studies Focused on BALF and Bronchial Aspirate Exosomal miRNAs

Only a handful of studies have evaluated airway-proximal exosomal miRNAs in lung cancer (Table 1):

### 5.3. Interpretation of Current Evidence

Despite the rapidly expanding literature on liquid biopsy in lung cancer, evidence specifically addressing exosomal miRNAs in airway-proximal fluids such as bronchoalveolar lavage fluid (BALF) and bronchial aspirate remains limited. This reflects both the procedural complexity of sample acquisition and the historical focus on blood-based biomarkers. Nevertheless, the available studies provide compelling proof-of-concept data supporting the biological and clinical relevance of this compartment.

One of the earliest studies directly investigating exosome-enriched miRNAs in BALF was conducted by Kim et al. (2018), who analyzed BALF-derived exosomes from patients with early-stage lung adenocarcinoma and benign pulmonary nodules [29]. Using ultracentrifugation-based isolation followed by quantitative PCR, the authors demonstrated significantly elevated levels of miR-126 and let-7a in cancer patients. Importantly, these miRNAs achieved area under the curve (AUC) values in the range of approximately 0.83–0.86, supporting good diagnostic performance in this setting. This work was pivotal in establishing that BALF exosomes are not merely detectable, but may offer enhanced diagnostic discrimination in early-stage disease, where circulating tumor DNA is often undetectable.

More recently, Huang et al. (2023) provided mechanistic and translational insights by identifying miR-1246b in BALF extracellular vesicles as a discriminator between malignant and benign pulmonary nodules [30]. Beyond diagnostic performance, this study linked miR-1246b expression to tumor-promoting signaling pathways, notably through fibroblast growth factor 14 (FGF14), and demonstrated associations with immune-suppressive features of the tumor microenvironment. This dual diagnostic and biological relevance strengthens the argument that airway-derived exosomal miRNAs reflect active tumor biology rather than passive shedding.

Evidence from bronchial aspirate and lavage samples, although less frequently enriched for exosomes, further supports the airway compartment as a biomarker reservoir. Rehbein et al. (2015) analyzed extracellular miRNAs in bronchoalveolar lavage samples from patients with lung cancer and benign lung diseases, identifying distinct expression patterns associated with malignancy [31]. While this study did not apply exosome-specific isolation methods, limiting direct comparability with more recent MISEV-aligned research, it demonstrated that the airway milieu contains disease-specific miRNA signatures and provided early support for the diagnostic relevance of airway-derived extracellular RNA.

Additional indirect evidence comes from studies investigating pleural lavage or pleural effusion-derived extracellular vesicle-associated miRNAs. Roman-Canal et al. (2019) identified diagnostic EV-miRNA panels in pleural lavage samples from patients with lung cancer, particularly in locally advanced disease [32]. Although pleural fluids reflect a later disease stage and different biological context, these findings reinforce the concept that tumor-adjacent fluids are enriched for biologically informative extracellular vesicles, lending further support to the investigation of BALF and bronchial aspirate in earlier disease settings.

Across the available studies, several consistent observations emerge. First, airway-proximal fluids demonstrate higher tumor specificity compared with peripheral blood, likely due to reduced dilution and closer proximity to the tumor microenvironment. Second, enrichment for extracellular vesicles improves the signal-to-noise ratio of miRNA detection, helping distinguish cancer-associated signatures from background inflammatory signals common in chronic lung disease. Third, the diagnostic performance reported for BALF exosomal miRNAs is comparable to, and in some cases exceeds, that reported for plasma-based liquid biopsy assays in early-stage lung cancer.

Nevertheless, the current evidence base is constrained by small sample sizes, heterogeneous patient populations, and substantial methodological variability. Differences in BALF instillation volumes, sampling sites, extracellular vesicle isolation protocols, and miRNA normalization strategies limit cross-study comparability. Furthermore, only a minority of studies explicitly adhere to MISEV2018 or MISEV2023 guidelines, raising concerns regarding extracellular vesicle purity, reproducibility, and reporting transparency [13,14].

In summary, although only a limited number of studies have directly evaluated exosomal miRNAs in BALF and bronchial aspirate, the available evidence consistently supports their diagnostic feasibility, biological relevance, and potential clinical value. These findings justify further investigation through well-designed, multicenter studies employing standardized airway sampling procedures and MISEV-aligned extracellular vesicle characterization protocols. Until such validation is achieved, airway-derived exosomal miRNAs should be regarded as promising but investigational biomarkers, positioned at the interface between conventional bronchoscopy and emerging molecular diagnostics.

## 6. Comparative Overview of Biofluids for Exosomal miRNA Analysis

A comparative overview of the main biofluids used for exosomal miRNA analysis in lung cancer is presented in Table 2. These matrices differ in terms of accessibility, proximity to the tumor, extracellular vesicle yield, and level of clinical validation, which directly influence their diagnostic applicability.

Plasma and serum are the most widely used due to their accessibility and established clinical workflows, although they may suffer from dilution of tumor-derived signals [17,19]. In contrast, airway-proximal samples such as bronchoalveolar lavage fluid (BALF) and bronchial aspirate offer higher tumor specificity due to their direct contact with the tumor microenvironment, albeit at the cost of increased invasiveness and lower standardization [33].

Other matrices, including sputum and pleural effusion, provide additional complementary information but are limited by variability in sample quality or applicability to specific disease stages [34].

## 7. Conceptual Workflow for Airway Exosomal miRNA Diagnostics–from Bronchoscopy to Molecular Diagnostics

The implementation of airway-derived exosomal miRNA analysis as a diagnostic tool in lung cancer requires a structured and reproducible workflow that integrates seamlessly into existing bronchoscopy-based clinical pathways. A conceptual framework encompassing standardized sample acquisition, processing, extracellular vesicle (EV) isolation, miRNA analysis, and clinical integration is outlined below.

### 7.1. Sample Collection

The workflow begins with bronchoscopic acquisition of airway-proximal fluids, most commonly bronchoalveolar lavage fluid (BALF) or bronchial aspirate. BALF is obtained by instilling sterile isotonic saline into a targeted bronchopulmonary segment, followed by gentle aspiration, while bronchial aspirate or washing samples are collected without saline instillation, reflecting proximal airway secretions. Sampling should be anatomically targeted to the radiologically or bronchoscopically suspicious region whenever feasible, as proximity to the lesion may influence EV concentration and tumor specificity.

Because bronchoscopy is already part of routine diagnostic evaluation for suspected lung cancer, incorporation of BALF or bronchial aspirate collection does not introduce additional procedural risk. Pre-analytical variables, including instilled volume, dwell time, suction pressure, and collection device, should be standardized and carefully documented, as these parameters significantly affect EV yield and downstream analytical performance.

### 7.2. Sample Processing and Pre-Analytical Handling

Following collection, airway samples should be processed promptly to preserve EV integrity and RNA stability. Initial low-speed centrifugation steps are used to remove cells, mucus, and debris, followed by higher-speed clarification to eliminate larger particles. Filtration through defined pore-size membranes may be applied to further reduce contamination by cellular fragments.

Time to processing is a critical determinant of sample quality. Delays beyond a few hours can lead to vesicle degradation and RNA fragmentation, potentially compromising reproducibility. Where immediate processing is not feasible, standardized short-term storage conditions should be employed. Detailed recording of pre-analytical handling steps is essential to ensure traceability and compliance with MISEV2023 recommendations.

### 7.3. Exosome and Extracellular Vesicle Isolation

Isolation of exosomes or small EVs from processed airway fluids represents a key analytical step. Ultracentrifugation remains the most widely used reference method, particularly in research settings, due to its ability to enrich small vesicles with acceptable purity. However, it is labor-intensive and operator-dependent.

Alternative approaches, including size-exclusion chromatography (SEC), polymer-based precipitation, immunoaffinity capture, and emerging microfluidic platforms, are increasingly explored for BALF and bronchial aspirate. SEC offers improved purity and preservation of vesicle integrity, while immuno-capture and microfluidic technologies enable higher specificity and scalability, making them attractive candidates for clinical translation. Regardless of the method used, EV preparations should be characterized using at least two complementary techniques to confirm vesicle size, concentration, and marker expression, in accordance with international guidelines.

While ultracentrifugation is widely considered the gold standard for extracellular vesicle isolation in research settings, its applicability in routine clinical laboratories remains limited. The requirement for specialized equipment, high operational costs, long processing times, and technical expertise restricts its use outside specialized centers [13,26].

In contrast, alternative methods such as size-exclusion chromatography (SEC) and polymer-based precipitation offer more practical and scalable solutions for clinical implementation. SEC provides improved reproducibility and preserves vesicle integrity, although it may result in lower yield [26,35]. Precipitation-based methods are user-friendly and do not require advanced equipment, but may co-isolate non-vesicular contaminants [36].

Emerging technologies, including immunoaffinity-based capture and microfluidic platforms, represent promising approaches for clinical translation. These methods allow for rapid processing, reduced sample volume requirements, and potential automation, making them attractive candidates for integration into routine diagnostic workflows [37,38].

Overall, the choice of isolation method should balance purity, yield, reproducibility, and feasibility, particularly in the context of future clinical implementation.

Beyond isolation, downstream analytical steps such as RNA extraction and normalization represent additional sources of variability that critically impact the reliability of EV-miRNA-based diagnostics.

### 7.4. RNA Extraction and miRNA Quantification

Following EV isolation, total RNA enriched for small RNA species is extracted using validated protocols optimized for low-input samples. Quantification of miRNAs may be performed using quantitative reverse transcription PCR, digital PCR, or next-generation sequencing, depending on the study aim and available infrastructure.

Normalization remains a major analytical challenge in airway-derived samples, as no universally accepted endogenous reference miRNA has been established for BALF or bronchial aspirate. Current best practices include the use of synthetic spike-in controls, global mean normalization, or carefully validated reference panels. Analytical validation should address sensitivity, linearity, reproducibility, and inter-assay variability, particularly if assays are intended for clinical decision-making.

Accurate quantification of EV-associated miRNAs requires robust and standardized analytical approaches. However, normalization remains a major challenge, particularly in airway-derived samples such as BALF and bronchial aspirate.

Unlike plasma-based assays, where several candidate reference miRNAs have been proposed, no consensus endogenous control has been validated for airway samples. Current strategies include the use of synthetic spike-in controls, global mean normalization, or selection of stable endogenous miRNAs based on experimental datasets [24,39].

Each approach has important limitations. Spike-in controls correct for technical variability but do not account for biological variation. Global mean normalization assumes stable overall miRNA expression, which may not hold true in heterogeneous clinical samples. Endogenous controls require prior validation and may vary depending on disease state and sample type [28,40].

In addition, pre-analytical variables—including sample dilution, mucus content, cellular contamination, and variability in EV yield—can significantly influence miRNA quantification and reduce reproducibility across studies [13,41].

**Figure 4:**
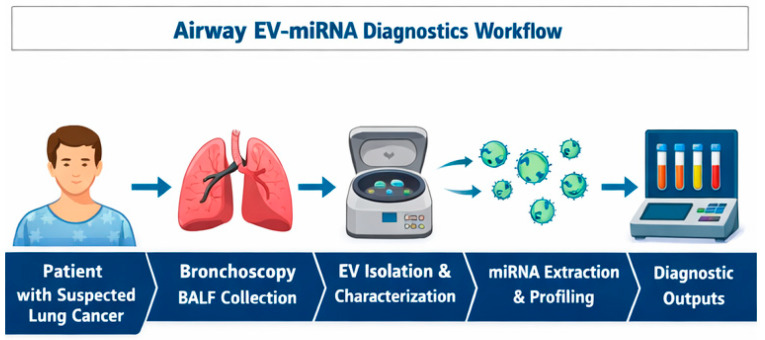
Airway EV-miRNA diagnostics workflow. BALF–bronchoalveolar lavage fluid; EV–extracellular vesicles; miRNA–microRNA.

These challenges highlight the urgent need for standardized normalization strategies and validated reference materials before EV-miRNA-based assays can be reliably implemented in clinical practice (Figure 4).

### 7.5. Toward Clinical Implementation

The final step of the workflow involves integration of exosomal miRNA data with existing diagnostic information, including imaging findings, cytology, histopathology, and clinical risk factors. Rather than serving as a standalone diagnostic test, airway exosomal miRNA analysis is best positioned as a complementary molecular layer that enhances diagnostic confidence and risk stratification.

In practical terms, EV-miRNA signatures could be used to refine the assessment of indeterminate pulmonary nodules, support malignancy likelihood estimates following non-diagnostic bronchoscopy, or contribute to longitudinal monitoring in selected clinical contexts. Integration into multivariable prediction models or decision-support algorithms may further improve clinical utility and facilitate adoption into routine practice.

For this conceptual workflow to progress toward clinical implementation, harmonization across centers is essential. Standard operating procedures covering sample collection, processing, EV isolation, and miRNA analysis must be established and validated in multicenter settings. In parallel, regulatory considerations, including analytical validation, quality control, and cost-effectiveness, must be addressed.

Importantly, the airway exosomal miRNA workflow aligns naturally with bronchoscopy-based diagnostics, offering a realistic pathway for translation in health systems where access to advanced imaging or repeated tissue biopsies is limited. With appropriate standardization and validation, this approach has the potential to transform bronchoscopy from a predominantly morphological procedure into an integrated molecular diagnostic platform.

Steps and interpretation as described are supported by reports of bronchoscopy-based EV sampling and microfluidic assay development [22,26,27,42].

## 8. Discussion

### 8.1. Diagnostic Implications

Airway-proximal fluids such as BALF and bronchial aspirate provide a direct molecular window into the lung tumor microenvironment. Because exosomes are released by both tumor and stromal cells, these fluids accumulate a concentrated signal of local pathophysiological activity [29,30,31,32]. Their use can significantly augment cytology, which shows variable sensitivity (50–70%) in small or peripheral lesions [43].

Importantly, bronchoscopy is already standard practice for evaluating suspicious lesions; no additional procedural risk is introduced when samples are also analyzed for EV-miRNAs [44,45]. Integrating such assays could convert a traditionally descriptive procedure into a quantitative molecular test, bridging cytology and precision genomics.

Combined interpretation of LDCT, cytology, and airway EV-miRNA panels could markedly reduce false positives and unnecessary biopsies. Early modeling studies indicate that adding a validated biomarker with AUC > 0.8 to LDCT screening could cut false-positive referrals by 25–30% [46].

From a clinical perspective, EV-miRNA signatures may be applied across multiple decision points. In screening settings, they could serve as adjuncts to LDCT to reduce false-positive rates. In diagnosis, they may support malignancy prediction in indeterminate pulmonary nodules, particularly when bronchoscopy yields inconclusive results. [46]

Furthermore, disease-specific EV-miRNA profiles may enable differentiation between histological subtypes (e.g., adenocarcinoma vs. squamous cell carcinoma) and potentially reflect molecular alterations such as EGFR or KRAS status. In longitudinal monitoring, dynamic changes in EV-miRNA expression could serve as indicators of treatment response or early relapse [43].

### 8.2. Prognostic and Predictive Potential

**Figure 5:**
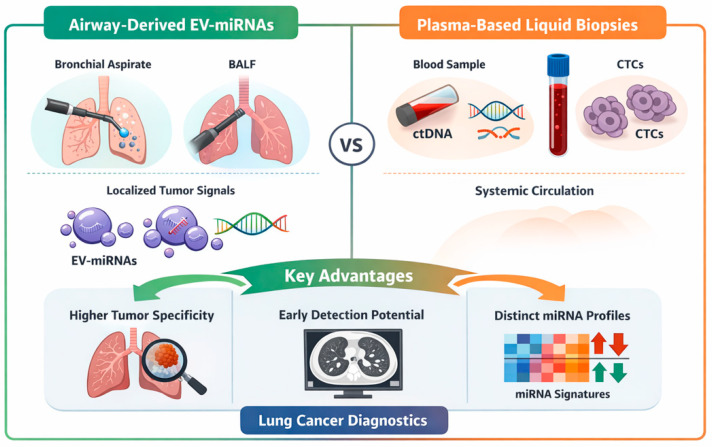
Schematic comparison of airway-derived EV-miRNA and plasma-based liquid biopsy.EV-miRNAs–extracellular vesicles microRNA. BALF–bronchoalveolar fluid; CTC–circulating tumoral cells; ctDNA–circulating tumoral DNA..

To integrate the key concepts discussed, a schematic diagram has been included illustrating the role of airway-derived EV-miRNAs within the broader liquid biopsy framework. This highlights the complementary nature of ctDNA, CTCs, and EV-miRNAs, as well as the specific advantages of airway-proximal sampling in capturing tumor-localized molecular signals (Figure 5).

The schematic summary illustrates the conceptual positioning of airway-derived EV-miRNAs within the broader liquid biopsy landscape. Compared with plasma-based biomarkers such as circulating tumor DNA (ctDNA) and circulating tumor cells (CTCs), airway-proximal samples—including bronchoalveolar lavage fluid (BALF) and bronchial aspirate—capture more localized tumor-derived signals due to their direct contact with the tumor microenvironment. This proximity results in higher tumor specificity and potentially improved diagnostic sensitivity, particularly in early-stage or localized disease. In contrast, blood-based biomarkers reflect systemic tumor burden but may be diluted and less sensitive in early stages. Importantly, EV-miRNAs from airway samples exhibit distinct molecular signatures that may enable more accurate discrimination between malignant and benign conditions, as well as between different tumor subtypes. Together, these complementary approaches highlight the potential of integrating airway-derived EV-miRNAs with established liquid biopsy modalities to enhance diagnostic accuracy and support personalized lung cancer management.

Specific exosomal miRNAs are intimately linked with cancer progression. miR-21 drives EMT and chemoresistance via PTEN/AKT signaling, miR-126 regulates angiogenesis, while miR-1246 and let-7a modulate immune evasion and metastatic potential [19,33,47,48]. Their presence in BALF or bronchial aspirate could therefore mirror tumor aggressiveness.

Longitudinal profiling of these miRNAs might help track treatment response or minimal residual disease. For example, decreasing exosomal miR-21 after surgery correlates with relapse-free survival in plasma studies [34]; the same principle could apply to airway fluids for local recurrence monitoring. Prospective validation is needed before clinical integration.

Beyond the most frequently reported miRNAs such as miR-21, miR-126, and let-7a, a broader spectrum of EV-associated microRNAs has been implicated in lung cancer biology. For instance, miR-155 has been associated with tumor-associated inflammation and proliferation [49], miR-210 is linked to hypoxia-driven tumor adaptation and metastatic signaling [50], and miR-486 has been correlated with tumor proliferation and survival pathways [19,51].

### 8.3. Methodological Challenges

Heterogeneity across sample collection protocols—instilled volume, dwell time, suction pressure, and anatomical site—can significantly influence EV yield [52]. Delay in processing leads to vesicle degradation and RNA fragmentation [36]. Uniform SOPs and pre-analytical documentation, as mandated by MISEV2023, are essential [13].

On the analytic side, different isolation techniques produce variable EV purity. Ultracentrifugation remains the reference, but size-exclusion chromatography and polymer precipitation are increasingly used for BALF [25,53]. Newer microfluidic devices provide high specificity and reproducibility, enabling potential point-of-care testing [39].

Normalization during miRNA quantification remains problematic because no stable endogenous reference exists for airway matrices. Synthetic spike-ins and global mean normalization are recommended interim solutions [28,39].

### 8.4. Romania’s Context and Opportunities

The primary goal of this section is to contextualize how airway-derived EV-miRNA diagnostics could be realistically implemented within the Romanian healthcare system. In particular, this approach leverages existing bronchoscopy infrastructure to introduce molecular diagnostics without requiring extensive additional resources.

Romania has one of the highest lung-cancer mortality rates in Europe, largely because 60–70% of cases present at stage III/IV [2,3,54]. Access to molecular testing (EGFR, ALK, ROS1) is limited outside tertiary centers, and national screening programs are nascent [4]. However, the National Plan for Beating Cancer 2023–2030 prioritizes early detection and precision diagnostics [4].

Given that bronchoscopy is routinely performed in pneumology centers, implementing airway EV-miRNA analysis could be cost-neutral, leveraging existing workflows. Multicenter Romanian research networks could collaborate to build standardized biobanks of BALF and bronchial aspirate, fostering regional leadership in exosomal biomarker validation [55,56].

Thus, airway EV-miRNA analysis represents not only a scientific opportunity but also a pragmatic strategy to bridge the gap between conventional bronchoscopy and precision oncology in resource-constrained settings.

### 8.5. Future Research Priorities

To facilitate a clearer overview of the key steps required for clinical translation, future research priorities in this field are summarized in the following table (Table 3) [13,19,33,42,47,48,57,58,59,60]. Given the early-stage nature of this field, a structured overview of research priorities is particularly valuable for guiding future multicenter and translational efforts.

Collectively, these priorities outline the necessary steps to transition airway-derived exosomal miRNAs from exploratory biomarkers to validated and clinically applicable tools integrated into guideline-based lung cancer management.

### 8.6. Limitations of Current Evidence

The evidence base remains sparse, dominated by small exploratory studies (n < 50) with heterogeneous methodologies [29,30,31,32]. External validation is absent, and publication bias likely inflates diagnostic accuracy. Until multicenter, MISEV-aligned studies emerge, airway EV-miRNAs should be regarded as investigational but biologically compelling biomarkers deserving continued research [13,53,57]. 

From an oncological perspective, airway-derived EV-miRNAs should not be viewed as standalone diagnostic tools, but rather as complementary biomarkers that can enhance existing clinical pathways. Their greatest potential lies in scenarios where conventional methods are inconclusive, such as indeterminate nodules or non-diagnostic bronchoscopy.

However, despite promising early data, their clinical adoption remains contingent on rigorous validation, standardization, and demonstration of added value beyond established biomarkers. Until such evidence is available, EV-miRNAs should be considered investigational tools with high translational potential rather than ready-to-implement clinical assays.

## 9. Conclusions

Airway-proximal fluids such as bronchial aspirate and BALF hold substantial promise as sources of exosomal miRNAs for early lung-cancer diagnosis and potentially prognostic evaluation. Current data, though limited, demonstrate feasibility and strong biological rationale. For Romania, integrating EV-miRNA analysis into bronchoscopy workflows could enhance diagnostic precision within existing infrastructure. Future multicenter trials adhering to MISEV2023 standards are crucial for clinical translation and international validation.

Author contributions: DH, CEB and GJ were responsible for conceiving the study design and methodology, drafting the manuscript and reviewing the manuscript, providing feedback as well as administration of the research team. DCS, HKS and MAV were responsible for drafting the manuscript and the database search. ESI and MBI were responsible for study analysis of eligible studies and drafting the manuscript.

## Figures and Tables

**Table 1:** Relevant studies included in the review.

Study	Year	Sample Type	Ev Isolation Method	MiRNAs Identified	Key Findings	Clinical Relevance	Limitations
Kim J.E. et al. [29]	2018	BALF exosomes	Ultracentrifugation + characterization (CD63, CD81)	miR-126, let-7a	Significantly higher expression in lung adenocarcinoma vs. controls; good diagnostic performance (AUC ~0.83–0.86)	Potential diagnostic biomarkers for early-stage lung cancer	Small sample size; limited validation cohort
Huang J. et al. [30]	2023	BALF-derived EVs	EV isolation (method consistent with EV studies; not fully standardized)	miR-1246	Promotes tumor progression via FGF14 signaling; diagnostic and mechanistic role	Biomarker + functional relevance in tumor biology	Limited clinical validation; single-center study
Rehbein G. et al. [31]	2015	BALF (total miRNA, not EV-specific)	Not EV-specific (whole BALF analysis)	Multiple miRNAs (panel)	Differentiates lung cancer from benign lung disease	Early evidence supporting BALF miRNAs as biomarkers	Lack of EV isolation; pre-MISEV standardization
Roman-Canal B. et al. [32]	2019	Pleural lavage EVs (not BALF, but airway-related)	EV isolation (ultracentrifugation-based)	miRNA panel (multiple)	Diagnostic discrimination in advanced lung cancer	Supports EV-miRNA utility in thoracic fluids	Not BALF-specific; advanced-stage patients

**Table 2:** Comparative overview of biofluids for exosomal miRNA analysis.

Matrix	Accessibility	Proximity to Tumor	EV Yield	Advantages	Limitations	Clinical Maturity
Plasma/Serum	Simple venipuncture	Low–moderate	Moderate	Widely used, repeatable	Diluted tumor signal	High
BALF/Bronchial aspirate	Bronchoscopy	High	High	Tumor-enriched, adjunct to cytology	Invasive, needs standardization	Early
Sputum	Non-invasive	Moderate	Variable	Easy, repeatable	Heterogeneous quality	Early
Pleural effusion/lavage	Thoracentesis/surgery	High	High	Reflects advanced disease biology	Limited to metastatic cases	Moderate

EV—extracellular vesicles; BALF—bronchoalveolar lavage fluid.

**Table 3:** Future research priorities.

Priority	Description	Clinical Relevance
Prospective multicenter trials	Recruitment of patients undergoing bronchoscopy for suspected lung cancer or indeterminate nodules, with standardized BALF/BAS collection and MISEV-aligned EV isolation protocols	Essential for validation, reproducibility, and generalizability of findings
Comparative matrix studies	Evaluation of diagnostic performance across biofluids (airway fluids vs. plasma vs. sputum)	Clarifies added value of airway-derived biomarkers over blood-based liquid biopsy
Mechanistic studies	Investigation of links between exosomal miRNAs and tumor biology, histology, and molecular subtypes (e.g., EGFR, KRAS)	Supports biological plausibility and potential predictive applications
Multi-omics integration	Combination of EV-miRNAs with ctDNA, proteomics, and metabolomics for composite biomarker models	Improves diagnostic accuracy and supports precision oncology approaches
Health-economic analyses	Evaluation of cost-effectiveness within screening and diagnostic programs, particularly in resource-limited settings	Critical for implementation in real-world healthcare systems
Standardization and regulatory readiness	Development of SOPs, analytical validation (precision, sensitivity, reproducibility), and reference materials	Required for regulatory approval and clinical adoption

## Data Availability

No new data were created or analyzed in this study.
